# After the fall: Responding to the Champlain Towers building collapse

**DOI:** 10.3389/fpubh.2022.1104534

**Published:** 2023-01-09

**Authors:** Deborah C. Beidel, David C. Rozek, Clint A. Bowers, Amie R. Newins, Victoria L. Steigerwald

**Affiliations:** ^1^UCF RESTORES, University of Central Florida, Orlando, FL, United States; ^2^Department of Psychology, University of Central Florida, Orlando, FL, United States

**Keywords:** posttraumatic stress disorder, first responders, disaster, depression, generalized anxiety

## Abstract

In June 2021, a condominium in Florida collapsed, with the loss of 98 lives. Search and rescue teams spent 2 weeks, recovering the victims. This study's objective was to assess the presence of psychological symptoms that might emerge in the following months, using the PTSD Checklist for DSM-5 (PCL-5), Patient Health Questionnaire−9 (PHQ-9), Generalized Anxiety Disorder – 7 (GAD-7), Suicide Cognitions Scale-Short (SCS-S), and the Insomnia Severity Index (ISI). A monthly survey conducted for 3 months found that overall, mean scores on these measures did not indicate significant emotional distress. We then compared the scores when the group was divided into responders who recovered human remains and those who did not. Scores were significantly higher among the subgroup that recovered human remains. Fifty-three percent (53%) of this sub-group met the cut-off score for a provisional diagnosis of PTSD, depressive disorder or generalized anxiety disorder−15% met the cut-off score criteria on the PCL-5 for probable PTSD, 36.8% for probable depressive disorder on the PHQ-9, and 26.3% for probable generalized anxiety disorder on the GAD-7. The results are consistent with other investigations examining mental health after mass disasters. Specifically, not all first responders will develop emotional distress but certain recovery activities may put some responders at higher risk, with a percentage displaying psychological distress. The results emphasize the need to assess the impact of these events on the mental health of first responders and to consider strategies to prevent or mitigate the development of impairing psychopathology.

## Introduction

First responders are repeatedly exposed to traumatic events such as fires, natural disasters, and accidents (1). In addition, there are unique traumatic events such as mass shootings and infrastructure failures including a building or bridge collapse. Following any of these events, first responders may experience trauma-related symptoms (e.g., difficulty sleeping, nightmares, hyperarousal). For most individuals, these symptoms remit with time (typically within 1–2 months) (2) and do not result in the development of mental health disorders (2, 3). For example, 82% of rescue and recovery responders to the 2001 World Trade Center attack reported low to moderate trauma symptoms that never rose to the level of a posttraumatic stress disorder (PTSD) diagnosis throughout the nine-year study period (4). Despite this overall positive outlook, some individuals will develop mental health disorders or experience symptom worsening in the months after a traumatic event (2, 5, 6). Predicting and tracking mental health outcomes following response to a disaster may lead to the development and improvement of post-disaster prevention, mitigation, and intervention services for first responders.

On June 24^th^, 2021, first responders from across the state of Florida responded to the collapse of the 12 story Champlain Towers condominium in Surfside, Florida. This building collapse resulted in the 3^rd^ highest death count in United States history, excluding terrorist events (7). There were 136 people in the building at the time of the collapse; 98 people lost their lives and all 98 were recovered from the debris pile (7). Twenty-two million pounds of debris were moved by hand in 29 days (7). There were 394 Florida urban search and rescue (USAR) team members who participated in the recovery process (7). The unexpected collapse initiated a significant search and rescue/recovery effort involving long work hours, exposure to dangerous conditions, and extensive recovery of human remains.

Because of the uniqueness of the event, the severity of the destruction, and the knowledge that recovery of all the deceased would (a) take weeks, (b) occur in extreme heat, humidity, and daily thunderstorms, and (c) exert significant physical and mental stress, behavioral health resources were mobilized within 72 h of the collapse to address any immediate needs of first responders. At that time, a comprehensive behavioral health disaster plan was not in place, although prior lessons learned from mass casualty events guided initial efforts. In this paper, we present the comprehensive behavioral health response implementation and the results of a post-deployment survey examining sleep, anxiety, depression, suicide cognitions, and PTSD, using the ISI, GAD-7, PHQ-9, SCS-S, and PCL-5, respectively.

During the active search and recovery process, there was a mobilization of behavioral health support for the urban search and rescue (USAR) teams, including deployment of comfort dogs, chaplains, peer support specialists, and mental health professionals for a two-week period. Comfort dogs and were stationed around the site, making them available to anyone who wanted to interact with them. Chaplains and peer support specialists were the initial responders to emergency personnel in distress, providing opportunities for responders to discuss their stress or emotional responses to the recovery work. For example, some first responders did not want to leave the work site at the end of their shift, even though they were exhausted. They wanted to stay and continue the search. Peers and chaplains intervened at those times, reminding responders that (a) others would take their place and (b) exhaustion could lead to mental mistakes and physical injuries - neither of which would hasten the recovery of the victims. They were able to connect in their role as a peer or fire chaplain, who could best understand the responders' concerns. They did not, however, provide formal psychological intervention. Mental health clinicians were on site and served as a back-up resource when a higher level of psychological care was needed. In those instances, mental health clinicians provided psychological first aid to stabilize the responder and planned for any needed support, either on-site or when the responder was demobilized.

In addition to the presence of personnel, printed literature, and palm cards with QR codes were available throughout the site (meeting areas, food tents). Some literature listed “tips to de-stress” that could be used immediately. Cards with QR codes provided lists of mental health resources (e.g., suicide support lines, resources available in various parts of the state). Finally, at the time of demobilization, teams received a psychoeducational debriefing about the potential effects of trauma, trauma symptoms that might occur (including insomnia nightmares, suicidal ideation) and were given mental health resources available in their area.

Because symptoms of trauma do not always occur in the immediate aftermath of a traumatic event (1), the purpose of this study was to assess the presence of common mental health complaints (anxiety, depression, posttraumatic stress disorder and sleep disorders) that might occur in the months following the rescue operation. It was hypothesized that a subset of first responders would develop significant mental health symptoms, such as nightmares, flashbacks, high levels of anxiety, depressed mood, and suicidal ideation as a result of the recovery effort.

## Materials and methods

### Survey procedure

Because this survey was anonymous and conducted online, the University's IRB determined that the study was “human subjects research exempt from regulation.” An introductory email, containing a survey link was distributed to each of the 8 state-wide USAR leaders, who distributed the information to all task force members who responded to the collapse. Interested first responders read the survey explanation and if they wanted to participate, clicked a yes button to indicate consent. This button opened a study page where they provided name, telephone number and email address (in case of the presence of suicidal intent). They then created their unique study code. After creating their study code, they were directed to a separate Qualtrics survey to complete the study questionnaires (see below). The time to complete the survey was approximately 30 min.

The survey collected information on demographics, role in the search and recovery process, mental health symptoms, and current mental health service usage. Although asking about mental health information has not been shown to increase risk of suicide (8), we provided information on crisis resources (e.g., National Suicide Lifeline, Crisis Text Line) to all participants at the end of the survey. Participants had the opportunity to complete the survey monthly and received a $25.00 gift card for each survey that they completed.

### Assessment measures

In addition to demographic data (see Table 1), the following measures were included in the survey:

**Table 1:** Sample demographics.

	**Mean**
Age	42.4 (range 28–66)
	***n*** **(%)**
**Sex**	
Male	29 (69.0%)
Female	8 (19.0%)
Did not specify	5 (11.9%)
**Race/Ethnicity**	
Hispanic	5 (11.3%)
Other	7 (15.9%)
White	32 (72.7%)
**Receiving mental health treatment**	10 (23.8%)

**PTSD checklist for DSM-5** [PCL-5; (9)]. The PCL-5 is a self-report measure assessing the 20 PTSD symptoms as outlined in the DSM-5. This 20-item scale has participants rate their endorsement of these symptoms on a scale from (0) not at all to (4) extremely. A total symptom severity score (range 0–80) can be obtained by summing the scores for each of the 20 items. A score of 31–33 is often used as an indicator of probable PTSD (10).

**Patient health questionnaire−9** [PHQ-9; (11)]. The PHQ-9 is a 9-item self-report measure of depression symptoms. Participants rate each item on a 0 (not at all) to 3 (nearly every day) scale. Scores from the PHQ-9 have been shown to be reliable and valid (11, 12). A score of 5 indicates the presence of mild depression (11), whereas a score ranging from 8 to 11 appears to have the most acceptable diagnostic properties for major depressive disorders with no difference in pooled sensitivity and specificity (13). Although originally developed based on DSM-IV criteria, the specific symptoms included in diagnostic criteria for major depressive disorder criteria remained the same in the DSM-5, thus allowing the PHQ-9 to remain as a valid measure of DSM-5 depressive disorder (14, 15).

**Generalized anxiety disorder−7** [GAD-7; (16)]. The GAD-7 is a 7-item self-report measure of general anxiety symptoms. Participants rate each item on a 0 (not at all) to 3 (nearly every day) scale. Reliability and validity of scores from the GAD-7 has been established and a score of 8 or higher indicates probable generalized anxiety disorder (16, 17). Although originally developed based on DSM-IV criteria, the specific symptoms included in diagnostic criteria for generalized anxiety disorder criteria remained the same in the DSM-5, thus allowing the GAD-7 to remain a valid measure of DSM-5 general anxiety disorder (14, 18).

**Suicide cognitions scale-short** [SCS-S; (19)] is a 3-item short form of the SCS, developed to assess suicidal ideation and other suicide-related beliefs. Individuals rate their agreement with items on a scale from 1 (strongly disagree) to 5 (strongly agree). The short form is comprised of 3 items with the best coverage of the latent variable of the SCS and are items from the Suicide Cognition Scale-Revised (19–21). Higher scores reflect greater severity of suicide-related beliefs.

**Insomnia severity index** [ISI; (22)] is a 7-item self-report screening measure of insomnia that assesses both the severity and impact of insomnia symptoms over the past 2 weeks. Items are rated on a scale from 0 (no problem) to 4 (very severe problem), with higher scores reflecting greater severity of insomnia symptoms. A cutoff of 15 has been suggested to indicate the presence of clinically significant insomnia symptoms (16).

## Results

The survey was initiated in September 2021. After the third month, the number of participants who continued to complete the survey was too small for further analyses, thus limiting this study to 3 monthly data points. Demographic characteristics of the participants are presented in Table 1. Table 2 depicts characteristics of the responder's role in the search and rescue. Forty-two individuals completed at least one part of the survey and the specific number of individuals who completed each psychological assessment at each specific time can be found in Table 3.

**Table 2:** Responder characteristics.

**Responder characteristics**	**Frequency or mean**
**Leadership or administrative role**	
Yes	13 (29.5%)
No	23 (52.3%)
Did not indicate	8 (18.2%)
**Number of days on site**	9 (range 2–23)
**Relationship to victims of the collapse**	
Knew someone who lived in the building	0
Knew someone who died in the building	0
Knew someone who lost someone in the building	6
**Experiences during their deployment**	**% responded yes**
Rescued survivors	4 (9.8%)
Recovered bodies of deceased adults	20 (46.3%)
Recovered bodies of deceased children	9 (21.9%)
Observed bodies being moved to the medical examiner	28 (63.4%)
Yelled at by family members (rescue was “too slow”)	9 (21.9%)
Physically injured	5 (12.2%)

**Table 3:** Means and standard deviations on mental health measures.

	**Mean and (Standard deviation)**
**PHQ-9**	
Time 1 (*n* = 36)	4.32 (5.38)
Time 2 (*n* = 30)	4.36 (5.29)
Time 3 (*n* = 26)	4.16 (5.28)
**PCL-5**	
Time 1 (*n* = 37)	10.50 (12.08)
Time 2 (*n* = 30)	11.86 (12.31)
Time 3 (*n* = 26)	10.16 (11.44)
**GAD-7**	
Time 1 (*n* = 37)	3.24 (4.30)
Time 2 (*n* = 30)	4.20 (4.99)
Time 3 (*n* = 26)	3.24 (4.21)
**SCS–short form**	
Time 1 (*n* = 36)	4.19 (1.54)
Time 2 (*n* = 30)	3.97 (1.47)
Time 3 (*n* = 26)	3.77 (1.24)
**ISI**	
Time 1 (*n* = 36)	7.86 (5.86)
Time 2 (*n* = 30)	7.90 (6.54)
Time 3 (*n* = 26)	7.42 (6.10)

### Mental health symptoms

At the time of the initial survey, first responders were not reporting significant depression (mean score on the PHQ-9 was below the cut-off of 5 for mild depression) and this remained unchanged across the next two assessment periods. However, 19% (*n* = 7) did endorse enough symptoms (score of 8 or above) to suggest the presence of moderate depressive symptoms (13) and this percentage did not change across the three assessment time periods. Similarly, PCL-5 scores were low both at the initial assessment and across time, with 8% of the sample (*n* = 3) scoring above the cutoff score of 31 for a probable PTSD diagnosis. Scores on the GAD-7 were low and did not change across the 3 months of the survey; 5% of the sample (*n* = 2) had scores that were above the cutoff score (8 or higher) for the probable presence of generalized anxiety disorder. Mean scores on the SCS-S and the ISI were also low and not indicative of specific problems in these areas. See Table 3 for means and standard deviations of each assessment measure.

### Mental health symptoms among first responders who recovered human remains

All first responders were exposed to dangerous physical conditions, particularly during the first 10 days when a portion of the condominium tower was still standing but structurally unsound. However, as depicted in Table 2, approximately half of the sample was involved directly in the recovery of human remains (adult or child) from the debris pile. Thus, we divided the group into responders who recovered human remains and those who did not and conducted group comparisons. As depicted in Table 4, first responders who recovered human remains had significantly higher PCL-5 scores [*F*_(34)_ = 4.30, *p* < 0.05; Hedges *g* = 0.32], significantly higher PHQ-9 scores [*F*_(34)_ = 8.02, *p* < 0.01, Hedges *g* = 0.44], and significantly higher GAD-7 scores [*F*_(34)_ = 6.51, *p* < 0.025, Hedges *g* = 0.48]. There were no significant differences on the SCS-S or the ISI.

**Table 4:** Mental health symptoms among responders who recovered human remains.

	**Mean and standard deviation**		***p* value**
	**Recovered human remains (*****n*** = **19)**	**Did not recover human remains (*****n*** = **17)**	
PHQ-9	4.95 (6.73)	2.52 (3.02)	<0.05
PCL-5	12.63 (14.34)	8.65 (8.91)	<0.01
GAD-7	4.11 (5.28)	2.01 (2.19)	<0.025
SCS	3.83 (1.24)	4.53 (1.74)	ns
ISI	8.78 (6.64)	7.06 (4.93)	ns

### Presence of psychological disorders among responders who discovered human remains

Although mean scores of the subgroup who recovered human remains were low, all the first responders who had scores that exceeded established cut-offs suggesting the presence of clinically significant symptoms were in this subgroup. Specifically, on the PCL-5, 15.8% (*n* = 3) scored 31 or higher, suggestive of a provisional PTSD diagnosis. On the GAD-7, 26.3% (*n* = 5) scored above the cut-off of 8 or higher, suggestive of the presence of generalized anxiety disorder, and on the PHQ-9, 36.8% (*n* = 7) scored above the cut-off score of 8 or higher, suggestive of the presence of depressive disorder. Overall, 10 out of the 19 responders who recovered human remains (53%) met the inventory cutoff scores for a possible diagnosis of either PTSD, generalized anxiety disorder, or depression.

## Discussion

As the public comes to recognize the need to address the mental wellness of first responders, it is clear that their occupation places this group among those at the highest risk for the development of psychological disorders (1, 23). Although most data available, to date, address the psychological effects of the everyday traumatic events experienced by this group, it is necessary to also understand the impact of large scale and catastrophic events, such as mass violence and natural or man-made disasters. For example, a Category 5 hurricane struck the Florida Panhandle region in 2018, resulting in 16 deaths and over $25 billion in damages. Approximately 1 year later, a mental health survey of firefighters who resided in and/or responded to this event revealed that 24.6% of the sample experienced moderate to severe levels of depression, 14% of the sample experienced moderate to severe levels of anxiety, and 18.9% reported symptoms that exceeded the cut-off score for a probable diagnosis of PTSD (24). Therefore, these unique events also are accompanied by emotional distress and suggest a need to consider mitigation efforts to potentially prevent such negative outcomes.

The Champlain Towers condominium collapse represented a unique and catastrophic event, with the potential to negatively impact the urban search and rescue teams who responded to the site. Based on the knowledge of prior large-scale events (4, 24), the Florida State Fire Marshall deployed comfort dogs, chaplains, peer support specialists, and mental health clinicians to be on site and available to the task forces. Our survey data indicated that none of the responders were expressing suicidal ideation in the months after the event but 19% of the sample reported mild sadness. Because we did not have pre-event depression scores, we cannot say conclusively that the reported sadness was a specific result of the traumatic event. However, given the extensive loss of life in this event, experiencing mild to moderate sadness was probably consistent with the disaster's scope and the first responders' role (recovery of the individuals, including children, who perished in the collapse).

Also consistent with the previous literature, responders closest to the trauma (in this case, those who discovered and removed human remains) were at higher risk for the development of emotional distress. This subgroup reported statistically higher scores on measures of generalized anxiety disorder, depression and PTSD when compared to the other first responders. Furthermore, across all the responders participating in the survey, all who scored above the cut-offs on these measures were in the group who had recovered human remains. Overall, 53% of this subgroup met the established cut-off scores for possible PTSD, depression, or generalized anxiety disorder. Given that we did not have mental health data on the USAR teams prior to the collapse, we cannot conclude definitively that these elevated symptoms resulted from the recovery of human remains. However, none of the individuals in the other group reported elevated scores at any of the assessment points, as one might expect if these disorders were randomly distributed across the USAR team members. Therefore, even with the appropriate caveats, the results are consistent with several tenants of trauma response - not everyone who experiences a traumatic event is impacted equally and depending upon individual experiences, certain subgroups may be at higher risk or require additional services.

Our results are not consistent with the mental health outcomes of the firefighters in the Florida panhandle who experienced Hurricane Michael (22). Responders to the Champlain Towers collapse came from across the state and returned to their homes an average of 2 weeks later. They did not have continued visual reminders of the traumatic event and none of the survey respondents knew anyone who lived in or died in the building. In contrast, many of the Hurricane Michael responders were impacted personally as well as professionally (24). Their homes were damaged, family members may have been unemployed, and resources were scarce–in some cases, delaying rebuilding for months, if not years. The multiple impacts from the Hurricane Michael event could surely have created the higher distress levels in that group.

One limitation for this study is the number of individuals who participated in the survey. Although fewer first responders participated than was expected, the overall results are consistent with previous investigations (2, 3), suggesting that for most task force members, there was minimal emotional distress following their recovery operations at Champlain Towers. An important caveat to this conclusion is that the battery included a limited number of measures. For example, the current study used a brief measure of suicide that could be expanded in future investigations in order to understand more about risk related to suicide.

A second limitation is that we did not carefully track the number of contacts that the mental health team made with the task force members. Therefore, although we know that all groups of resources (peer support specialists, chaplains, comfort dogs, and mental health clinicians) were used, we did not monitor the frequency of the interactions. Having served as part of the group deployed, we know that the on-site mental health resources were utilized daily during the 2 week period. Did this immediate deployment prevent later emotional distress? That remains unclear but untangling these possibilities is worthy of further investigation. It is possible that the informal on-site engagements mitigated initial or later emotional distress, but without a controlled clinical trial, such a conclusion remains speculative. In summary, this investigation adds to the small but growing body of literature addressing the mental wellness of first responders in the wake of catastrophic events that result in the significant loss of life. The results suggest that 3 months after the event, most responders were not experiencing psychological distress. However, among the subset that recovered human remains, scores on measures of depression, anxiety and trauma were statistically significantly higher and for a percentage of that group, exceeded the cut-off scores indicative of the presence of potential psychological distress. Close monitoring of individuals who are exposed to the most horrific aspects of catastrophic events is needed, and perhaps more formal and expansive implementation of the informal support strategies (peer support, comfort dogs, chaplains, with mental health clinicians on stand-by) designed to mitigate the disaster's impact.

## Data availability statement

The raw data supporting the conclusions of this article will be made available by the authors, without undue reservation.

## Ethics statement

The study involving human participants was reviewed and approved by University of Central Florida Institutional Review Board. Written informed consent for participation was not required for this study in accordance with the national legislation and the institutional requirements.

## Author contributions

DB, DR, and AN contributed to the overall design of the study. CB conducted the data analysis. VS constructed the surveys. DB, CB, AN, and DR contributed to the construction of the final manuscript. All authors contributed to the article and approved the submitted version.
